# Management of a complicated crown root fractured tooth via intentional replantation with 180° rotation: a 4-year case report

**DOI:** 10.3389/fdmed.2026.1791938

**Published:** 2026-04-07

**Authors:** Jianxin Wang, Yongliang Wang, Kaijie Lin, Rui Han, Lei Ma

**Affiliations:** 1 Department of Prosthodontics, The Affiliated Hospital of Qingdao University, Qingdao, China; 2 School of Stomatology, Qingdao University, Qingdao, China; 3 Department of Stomatology, The Affiliated Hospital of Qingdao University, Qingdao, China

**Keywords:** crown-root fracture, follow-up study, tooth fractures, tooth replantation, traumatic dental injury

## Abstract

This case report describes the successful treatment and 4-year follow-up of a maxillary central incisor with a complicated crown–root fracture that had sustained two separate traumatic injuries during the transition from adolescence to adulthood. Following the initial trauma in adolescence, the tooth was treated with root canal therapy and reattachment of the fractured crown fragment. A second trauma in adulthood led to a complicated crown–root fracture whose margin extended subgingivally. The tooth was managed using intentional replantation with 180° rotation and subsequent restoration with an all-ceramic crown. After 4 years, clinical and radiographic examinations revealed a healthy outcome with no signs of root resorption or ankylosis.

## Introduction

Dental trauma is a significant public health concern, currently ranking as the fifth most common disease worldwide (1), with a permanent tooth trauma incidence of approximately 15% (2), and is widely recognized as one of the primary causes of tooth loss (3). The anterior teeth are particularly susceptible to dental trauma (4). Complex crown–root fractures represent a severe form of trauma, where the fracture originates from the crown and extends obliquely to the subgingival root region or even reaches the alveolar ridge. Given their frequent occurrence in the anterior region, preserving the fractured root while ensuring aesthetic restorability is a significant challenge. Restoring teeth with complex crown–root fractures presents multiple challenges: the restoration margin inevitably encroaches upon the biological width, leading to chronic inflammation and alveolar bone resorption; deep fracture lines can compromise marginal seal integrity; and postrestoration crown–root proportions become disproportionate. Common restoration approaches for complex crown–root fractures include (5) crown bonding, implant-supported restoration, orthodontic traction, crown lengthening, and intentional replantation. This last method involves extracting teeth that are difficult to preserve using conventional methods, performing external examinations and treatments, and then replanting them back into the extraction socket (6). Clinical studies (7) have indicated that the 4-year retention rate for intentionally replanted teeth is 82.8%, while another study (8) revealed that the 5-year retention rate for intentionally replanted teeth with apical periodontitis reaches 86.7%, demonstrating the reliability of this treatment approach.

Long-term reports on aesthetic restoration through 180°-rotated tooth replantation, particularly in cases involving secondary trauma spanning the patient's growth and development period, remain uncommon. This case report aims to describe the comprehensive management and successful experience of a patient whose tooth harboured a complex crown–root fracture resulting from two traumatic events during adolescence and adulthood, reporting its successful outcome at a 4-year follow-up after replantation. This study provides new insights into the comprehensive management and potential replantation of teeth with complex crown–root fractures in adolescents.

## Case report

### This case report was prepared in accordance with the CARE guidelines

A 16-year-old male with a fractured right maxillary central incisor (tooth 11) that had occurred one day prior due to trauma presented at our department. The patient had initially been seen at an emergency dental clinic, where the fractured crown fragment was temporarily reattached. Clinical examination revealed a horizontal fracture at the buccal cervical margin of tooth 11 with pulp exposure. The palatal portion of the crown remained partially intact but was mobile, whereas the root itself exhibited no notable mobility. Radiographic examination confirmed a cervical fracture line with no evidence of root fracture. After the treatment options were discussed with the patient and his parents, root canal treatment (RCT) was initiated, and the crown fragment was temporarily stabilized. Definitive reattachment using a fibre post and adhesive resin was planned following completion of the RCT, with the possibility of a new restoration after the patient reached adulthood.

**Figure 1:**
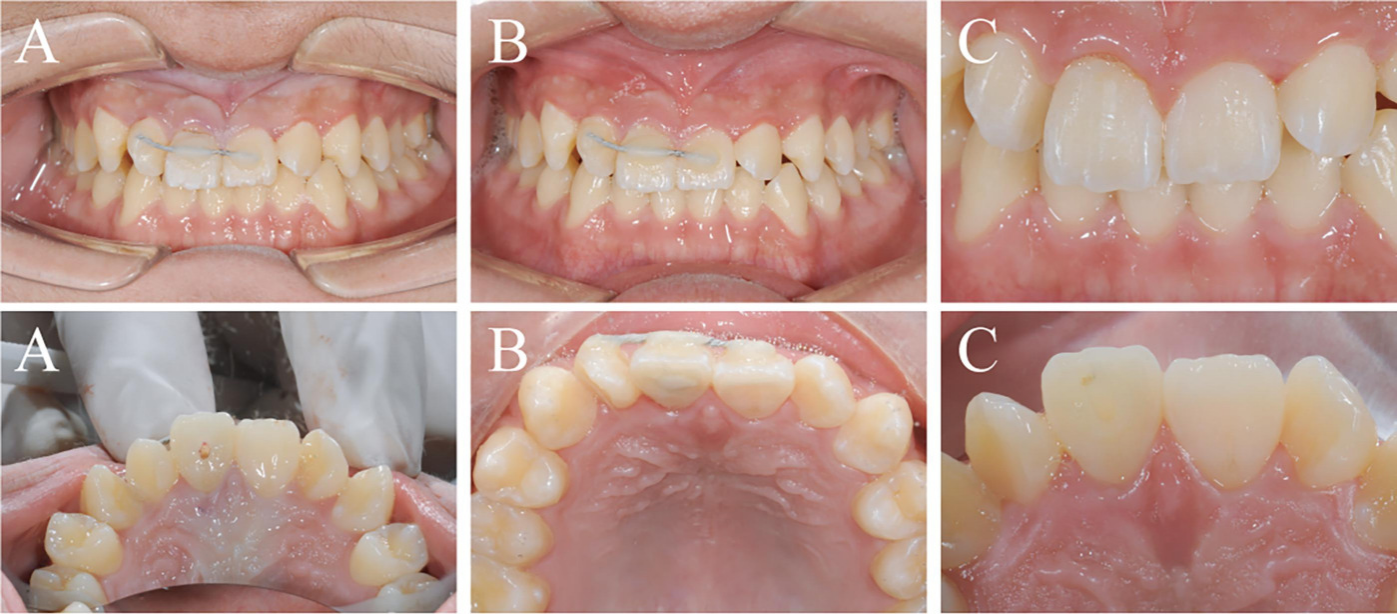
Oral images: **(A)** crown fragment stabilization; **(B)** following fiber post placement; **(C)** removal of the stabilizing splint.

**Figure 2:**
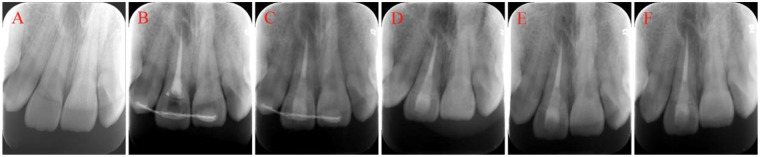
Serial periapical radiographs of tooth #11 demonstrating the treatment sequence: **(A)** initial presentation of the crown-root fracture; **(B)** post-obturation radiograph confirming adequate root canal filling; **(C)** radiograph after fiber post placement demonstrating coronal stabilization; **(D)** 3-month follow-up; **(E)** 6-month follow-up; **(F)** 12-month follow-up.

Under local anaesthesia, the fractured crown of tooth 11 was repositioned. The labial surfaces of teeth 12 to 21 were acid-etched, and the crown fragment was stabilized using flowable composite resin and a stainless-steel wire. An access cavity was prepared on the lingual surface of tooth 11. The pulp was extirpated, and root canal preparation was performed using nickel–titanium instruments. The canal was copiously irrigated with 3% NaOCl, dried, and dressed with calcium hydroxide paste. The access cavity was sealed with a temporary glass ionomer restoration. One week later, the intracanal medication was removed. After the final irrigation and drying, the root canal was obturated using gutta-percha and AH Plus sealer with the vertical condensation technique. Radiographic confirmation revealed adequate obturation. One week after RCT, a post space was prepared, and a fibre post was luted with resin adhesive to provisionally stabilize the crown fragment. Two weeks later, the stabilizing wire on teeth 12–21 was removed, and the surfaces were polished. Follow-up examinations at 3, 6, and 12 months revealed no symptoms. Radiographs revealed no signs of root resorption, ankylosis, or periapical pathosis (Figures 1, 2). (The detailed timeline of diagnosis and treatment is provided in Table 1).

**Table 1:** Timeline of treatment and follow-up for tooth #11.

Time Point	Key Event/Intervention	Clinical/Radiographic Findings
Age 16; Initial trauma	Fracture diagnosis; Temporary stabilization	Horizontal fracture with pulp exposure; No root fracture on radiograph
Subsequent treatment	Completion of RCT; Fiber post placement	Asymptomatic; Adequate obturation on radiograph
3-, 6-, 12-month follow-up	Clinical review	No symptoms; No pathology on radiograph
Age 18; Second trauma	Diagnosis of complicated crown-root fracture	Fracture 4 mm subgingivally palatally; 5 mm PD; CBCT confirmed fracture line
Day of second trauma	Intentional replantation with 180° rotation	Tooth atraumatically extracted, rotated, and replanted
1-week post-op	Splint removal	Grade I mobility; No resorption on radiograph
1-month post-op	Delivery of provisional crown	Tooth non-mobile; No percussion sensitivity; No resorption on radiograph
6-month post-op	Definitive all-ceramic crown delivery	Healthy gingiva; Normal probing depths; No radiographic abnormalities
1-, 2-, 3-, 4-year follow-up	Periodic review	Aesthetic gingival contour; Stable function; No signs of ankylosis, resorption, or bone loss

Two years later, the patient, now an adult, returned to our clinic following a new traumatic injury to the same tooth (tooth 11) one day prior. Clinical examination revealed a fractured crown with its buccal fracture margin at the gingival level and its palatal margin extending approximately 4 mm subgingivally. A 5 mm periodontal pocket was detected on the palatal aspect. The tooth was not tender to percussion. CBCT imaging revealed a fracture line located 14.5 mm from the apex. The palatal fracture margin was situated approximately 1 mm coronal to the alveolar bone crest. The root filling appeared satisfactory, with no periapical radiolucency or root fracture. Based on the clinical examination and CBCT findings, a diagnosis of a complicated crown-root fracture was confirmed. After a detailed discussion of all treatment options, risks, and benefits, the patient opted for intentional replantation with 180° rotation (buccolingual direction) in an effort to preserve the natural root.

Under local anaesthesia (1.7 mL of articaine with epinephrine), the root of tooth 11 was atraumatically extracted using minimally invasive instruments, with care taken to preserve the periodontal ligament and cementum. A sterile cotton pellet was placed in the socket to prevent contamination. The root was immediately inspected under a microscope for cracks while it was kept moist with saline. After confirming the absence of fractures, the root was rotated 180° in the buccolingual direction and replanted into its socket. The root length within the alveolar bone was adjusted to ensure adequate space for the final restoration, an appropriate crown-to-root ratio, and respect for the biological width. Splinting was performed using orthodontic wire and flowable composite resin on teeth 12 to 21. To address both aerobic and anaerobic oral bacteria that may be introduced during the surgical procedure, the patient was prescribed oral antibiotics (cefdinir 0.1 g every 8 h for 7 days; metronidazole 0.2 g every 8 h for 7 days) and an antiseptic mouth rinse to prevent infection. At the 1-week follow-up, the splinting wire on the labial surfaces of teeth 12–21 was removed. Tooth 11 exhibited grade I mobility. Radiographic examination revealed no radiolucent areas around the root.

**Figure 3:**
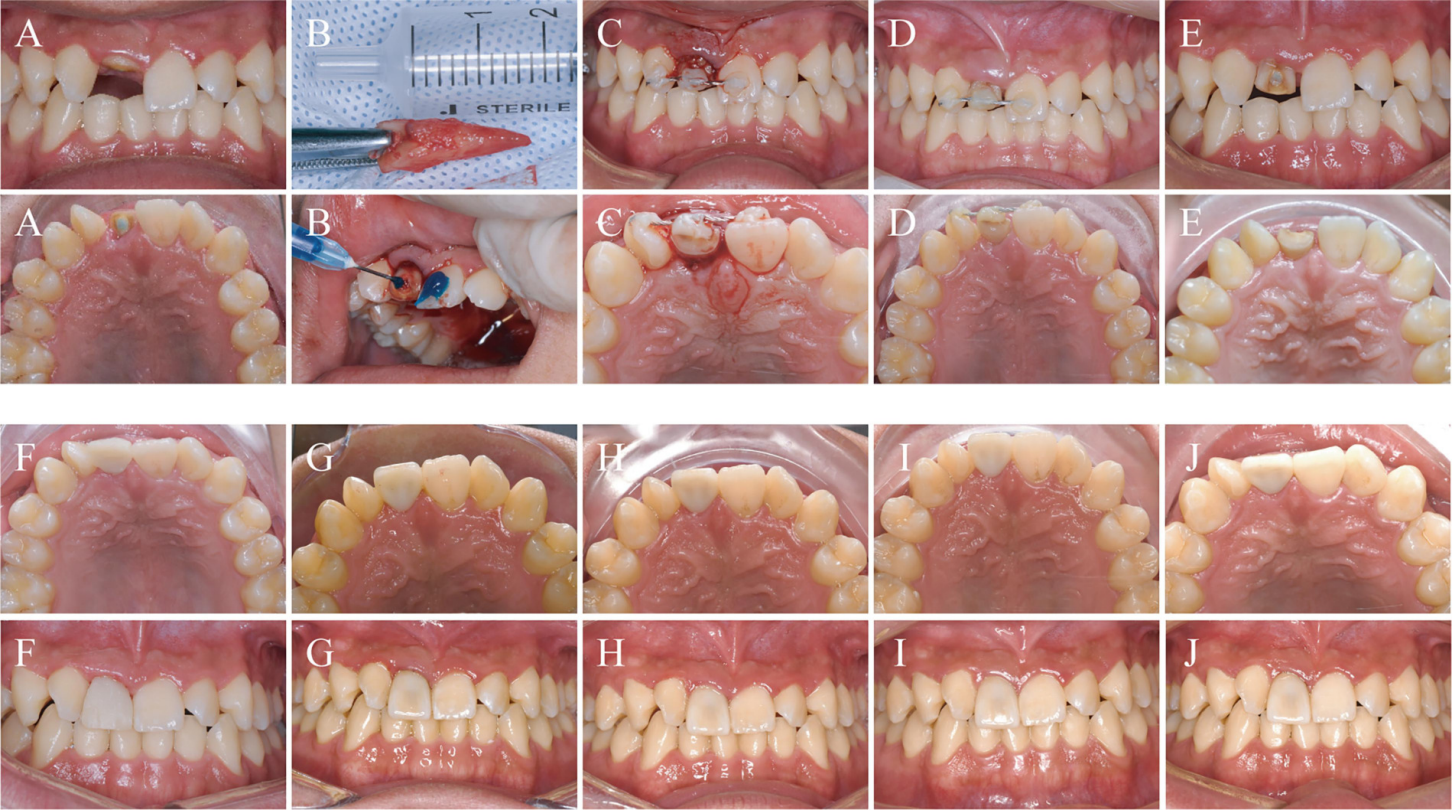
Oral images: **(A)** clinical appearance after the second trauma; **(B)** tooth extraction and intentional replantation; **(C)** splinting of the replanted tooth; **(D)** 7-day follow-up after replantation; **(E)** after splint removal; **(F)** provisional crown restoration; **(G)** definitive all-ceramic crown; **(H)** 1-year follow-up; **(I)** 2-year follow-up; **(J)** 4-year follow-up.

**Figure 4:**
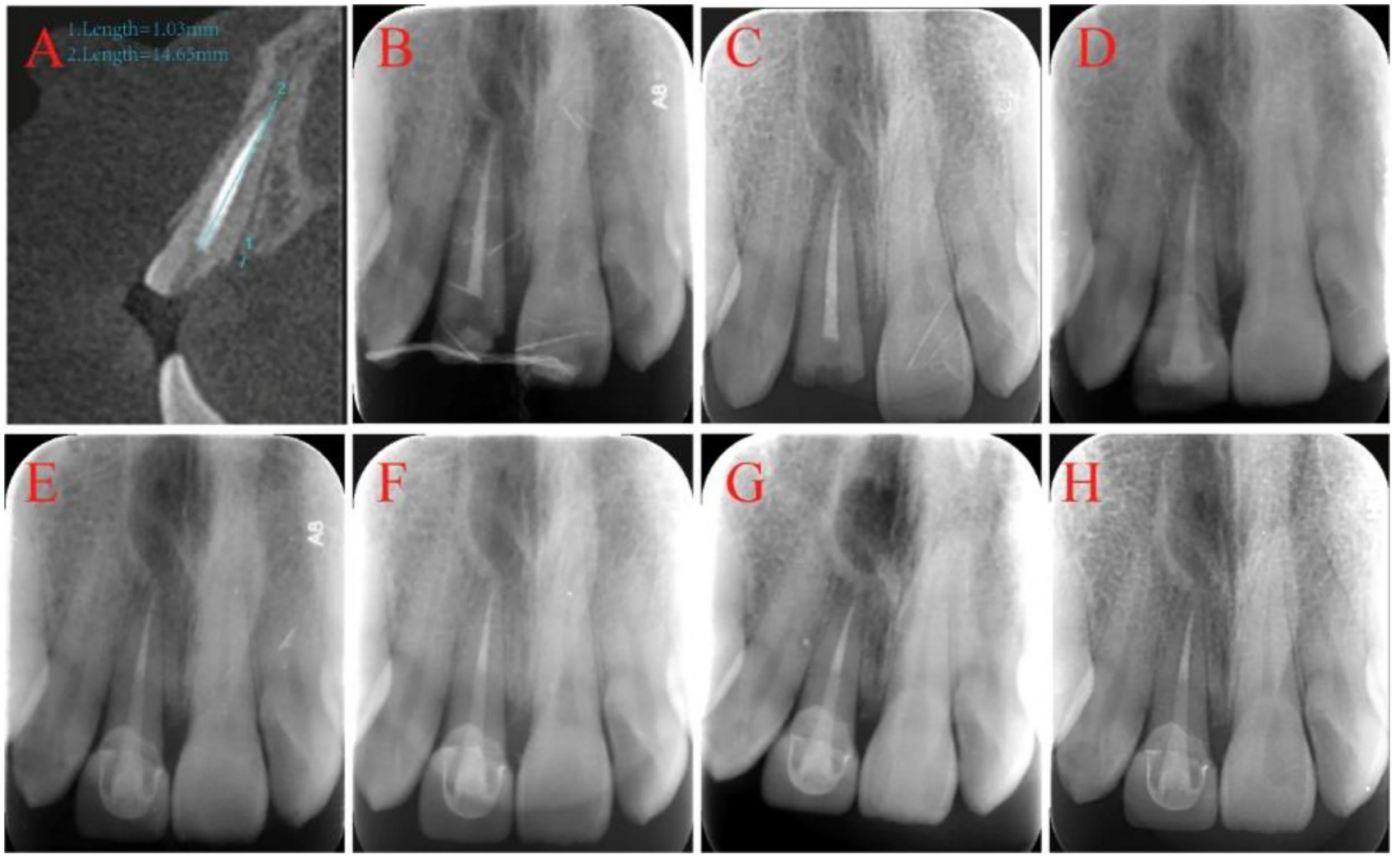
CBCT and radiographs image: **(A)** CBCT image after the second trauma; **(B)** post-intentional replantation; **(C)** 7-day follow-up; **(D)** 6-month follow-up; **(E)** 1-year follow-up; **(F)** 2-year follow-up; **(G)** 3-year follow-up; **(H)** 4-year follow-up.

One month postoperatively, the tooth was nonmobile and unresponsive to percussion. Radiographs revealed no evidence of resorption or periapical pathology. A post space was then prepared within the existing root canal. A fibre post was cemented with resin adhesive, a resin core was constructed, and a temporary crown was placed. After 6 months of temporary restoration with no clinical or radiographic abnormalities (no ankylosis, root resorption, or bone loss), the tooth was ultimately restored with an all-ceramic crown. At the 4-year follow-up, the gingival contours were aesthetically pleasing. Periodontal probing revealed probing depths ≤3 mm at all sites, with no bleeding on probing and no attachment loss. Radiographic examination revealed no signs of ankylosis, root resorption, or alveolar bone loss (Figures 3, 4).

## Discussion

This case of a complex crown–root fracture in an adolescent is notable for its unique combination of several clinically significant features: the sequential management of two traumatic injuries to the same tooth, a staged treatment approach transitioning from adolescence to adulthood, and the successful use of intentional 180°-rotation replantation to address a deep subgingival fracture, resulting in favourable clinical and radiographic outcomes over a 4-year follow-up period.

At the time of the first trauma, the patient was 16 years old with incomplete tooth growth. Studies indicate that passive tooth eruption may continue until 18–19 years of age, with potential vertical changes of up to 0.5 mm during this period (9). Performing a permanent restoration too early is associated with a risk of future crown margin exposure, disrupted proximal contact or occlusal harmony, and unpredictable gingival margin levels in the aesthetic zone. Similarly, implant therapy is generally not recommended for adolescents, as their continued jaw growth and soft tissue development may lead to malocclusion, unaesthetic gingival contours, or submergence of the implant relative to the adjacent natural teeth (10). Therefore, after the initial trauma, the treatment consisted of root canal therapy followed by reattachment of the fractured crown using a fibre post. Definitive restoration was postponed until adulthood.

Unfortunately, the tooth sustained a second traumatic injury two years later, with the palatal fracture margin now located 4 mm subgingivally. By this time, the patient had reached skeletal maturity. The treatment options offered included implant restoration, orthodontic extrusion, surgical crown lengthening, and intentional replantation with 180° rotation. Managing deep subgingival fractures in the aesthetic zone requires careful consideration of the available treatment options. Orthodontic extrusion can expose the fracture margin, but it requires a prolonged treatment duration, demands good patient compliance, carries a risk of relapse, and may alter the crown-to-root ratio, potentially affecting long-term stability (11). Surgical crown lengthening, while faster, inevitably sacrifices alveolar bone support, compromises periodontal tissues, and often results in aesthetic deficiencies in the anterior region. Implant restoration, though a viable option, was deferred in this young patient who strongly desired to preserve his natural tooth root and proprioception. In contrast, intentional replantation offered a single-visit solution that preserved the natural root and alveolar bone support while shortening the overall treatment time. This approach allows thorough extraoral inspection of the root for cracks and minimizes alterations to the gingival architecture, which is crucial for aesthetic outcomes. However, these benefits must be weighed against the technical sensitivity of the procedure and the potential risk of post-replantation root resorption.

Although no specific survival rates have been reported for 180°-rotation replantation, it is reasonable to postulate that its success rate might be lower than that of conventional intentional replantation, in which the root anatomy aligns precisely with the socket. A 180° rotation can introduce discrepancies between the root surface and the socket wall. Taking the maxillary central incisor as an example—which typically has a blunt, triangular cervical cross-section—rotation may create a gap in the cervical region that could compromise the reattachment of periodontal ligament fibres. Mild cervical resorption of the alveolar bone or root surface has been reported in cases by Yang et al. (12), Kim et al. (13), and Raj et al. (14). This resorption may be attributed either to minor trauma during the extraction procedure or to the presence of a gap resulting from the rotation. At present, the number of available cases is limited, and the clinical quantification of such gaps and their direct relationship with resorption remains challenging. Despite these minor complications, the overall outcomes have been satisfactory, suggesting that roots with a more conical shape may be better suited for this technique. To mitigate potential gaps, the local application of biocompatible materials such as platelet-rich fibrin (PRF) (15) or enamel matrix derivatives (emdogain) (16) at the replantation site should be considered.

Beyond simply relocating the fracture margin, the 180° rotation confers a specific biological advantage. The principle relies on repositioning a healthy, uninjured portion of the root surface (originally the buccal aspect) adjacent to the healthy socket wall in the critical subgingival palatal area. This creates an optimal environment for periodontal ligament (PDL) healing. As PDL cells possess the remarkable capacity to reorganize and reattach, placing viable PDL from the former buccal side against the palatal socket wall promotes the regeneration of a new functional attachment, effectively sealing the oral environment from the underlying bone. The success of replantation depends critically on the viability and regenerative capacity of periodontal ligament (PDL) cells (17). Protecting these cells helps prevent replacement resorption (ankylosis) (18). In this case, a minimally invasive extraction technique was employed at the cementoenamel junction to minimize damage to the PDL and cementum. The root was kept moist with saline during the extraoral period (19), rapidly inspected for cracks, and replanted with rotation within a short duration; this is considered a key factor for success, with 15 min often considered a critical timeframe (20). This technique is particularly suitable for younger patients because of their greater PDL healing potential (21, 22); the oldest patient reported to have undergone successful intentional 180° replantation was 30 years old at the time of surgery, who demonstrated a good outcome at the 1-year follow-up (20).

Pulp status also significantly influences the patient's outcome. Infection from necrotic pulp can lead to inflammatory resorption (18). In this case, uniquely, the tooth had already undergone complete root canal treatment before the second trauma, eliminating a potential source of infection. With respect to the timing of endodontic therapy, some protocols advocate for root canal treatment one week after replantation (13, 20), whereas in other protocols, as in the present case, root canal treatment is completed prior to replantation (14, 23). Although both approaches have achieved successful outcomes, performing thorough root canal treatment *before* replantation—as in the present study—may be considered more prudent. This strategy ensures the complete elimination of intracanal microbial contaminants in advance, thereby establishing optimal conditions for subsequent periodontal healing. Furthermore, calcium hydroxide is the preferred intracanal medication for traumatized teeth owing to its dual antibacterial and antiresorptive properties (24). These meticulous details are paramount for ensuring the long-term success of replanted teeth.

The patient's compliance throughout the treatment course was indispensable to a successful outcome. His strong preference for preserving the natural tooth was the decisive factor in selecting 180° rotational replantation over an implant-supported restoration. During the four-year treatment and follow-up period, the patient demonstrated excellent adherence to all postoperative instructions and oral hygiene protocols, which was crucial for uneventful healing. At the most recent follow-up, he expressed a high level of satisfaction with the final aesthetic outcome, including the gingival contour, tooth colour, and morphology.

In conclusion, this case involved the management of two separate traumatic injuries to the same tooth from adolescence through adulthood, culminating in 180°-rotation replantation. At the 4-year follow-up, the tooth was clinically asymptomatic and radiographically free of signs of ankylosis, root resorption, or bone loss. Studies have suggested that replanted teeth tend to stabilize at approximately 48 months post-operation (25). The treatment strategy was tailored to the patient's age and developmental stage at both trauma events, resulting in a successful outcome. Excellent patient compliance and meticulous oral hygiene were also indispensable contributors to this favourable result.

## Conclusion

This case report describes the successful management of a tooth that sustained two separate traumatic injuries between adolescence and adulthood. The complex crown–root fracture was ultimately treated using intentional replantation with 180° rotation, resulting in excellent aesthetic and functional outcomes and a favourable long-term result. In conclusion, for cases of complicated crown-root fractures, intentional replantation with 180° rotation represents a viable treatment option that has the potential to preserve the natural tooth structure while restoring both function and aesthetics. As a single case report, the findings of this study are not generalizable. The success of this technique is highly dependent on strict case selection, minimally invasive surgical procedures, comprehensive infection control protocols, and good patient compliance. The successful 4-year follow-up period presented in this case provides further evidence supporting the effectiveness of this technique.

## Data Availability

The raw data supporting the conclusions of this article will be made available by the authors, without undue reservation.
